# How strong are Malawi’s family planning programs for adolescent and adult women? Results of a national assessment of implementation strength conducted by Malawi’s National Evaluation Platform

**DOI:** 10.7189/jogh.09.020901

**Published:** 2019-12

**Authors:** Samuel Chipokosa, Anooj Pattnaik, NEP Malawi Technical Task Team, Amos Misomali, Diwakar Mohan, Michael Peters, Fannie Kachale, Jameson Ndawala, Melissa A Marx

**Affiliations:** 1 National Statistical Office, Zomba, Malawi; 2 Department of International Health, Johns Hopkins Bloomberg School of Public Health, Baltimore, Maryland, USA; 3 National Evaluation Platform, Malawi; 4 Reproductive Health Directorate, Ministry of Health, Lilongwe, Malawi; * Members of the NEP Malawi Technical Task Team is listed at the end of the article

## Abstract

**Background:**

To assess the strength of implementation of family planning programs targeting youth (15-24) in Malawi with a specific focus on youth and the Youth-Friendly Health Services program

**Methods:**

We conducted 9781 mobile phone interviews with facility in-Charge Nurses and health workers (health facility workers, health surveillance assistants [HSAs] and community-based distribution agent [CBDAs]) who provide family planning (FP) services across the 28 districts. Responses were entered in tablet using Open Data Kit. They were summarized and presented using R, Stata (College Station, TX, USA, StatsReport, JHU, Baltimore, MD, USA) and ArcView GIS (ESRI, Redlands, CA, USA).

**Results:**

Availability of key products was a challenge across all health worker types as only 39% of health facilities, 29% of HSAs and 45% of CBDAs had all the FP methods they are supposed to provide on the day of the interview. About 50% of health workers were supervised within past 90 days preceding the study. Despite most facilities saying that they provide youth friendly health services, youth-specific FP guidelines or protocols were not available in 43% of facilities that provide these services and only 33% of facilities had special rooms and 58% have special days for youth.

**Conclusions:**

The commodity supply system needs to ensure that all facilities and workers have a consistent supply of all contraceptive methods. Government and program implementers should ensure availability of all FP guidelines and information, education, communication materials at all service delivery points and facilitate creation of special rooms or days for youth.

Total fertility rates (TFRs) have declined substantially in many LMIC over the past two decades at the same time as some countries have reduced their child mortality rates [1]. The confluence of these factors has led to an increase in the number and proportion of young people, also known as a “youth bulge” [2]. Increased survival is indisputably positive. But as these children reach adulthood new population pressures require improvements in the effectiveness and efficiency of family planning (FP) programs, and an increased focus on youth [2,3].

Malawi is a small country in south central Africa, home to an estimated 18 million people occupying 118 484 km^2^, with the third lowest per capita GDP globally [4]. By 2020, 64% of Malawi’s population is projected to be under 25 years of age [5]. Projections estimating that the population would double from 2008 to 2030 prompted government officials to raise concerns about their capacity to provide services to the increased population [6]. Malawi’s TFR dropped from 5.7 to 4.4 from 2010-2015 (7, 8), below the 4.6 TFR used in population projections, so the rate of population growth is also projected to slow. But challenges remain. In 2015, over 59% of 19-year old women reported having begun childbearing, only a modest decrease from 2010 (64%) [7,8]. Even with declining TFR, in the context of a youth bulge, with such a high proportion of youth beginning childbearing we expect a continued and dramatic increase in need for FP support for youth [6].

The Government of Malawi recognized these needs a decade ago and in 2007 developed a Youth-Friendly Health Services (YFHS) program, “high-quality services that respond to the general health, especially sexual and reproductive health and rights (SRHR), needs of young people”[9]. YFHS are designed to be “relevant, accessible, attractive, affordable, appropriate, and acceptable to young people” (YFHS Strategy 2015-2020) and are implemented at facilities and through a semi-professionalized cadre of Health Surveillance Assistants (HSA) and a volunteer cadre of Community-Based Distribution Agents (CBDA). HSAs provide condoms, oral contraceptive pills (OCPs), and injectables and CBDAs provide condoms and OCPs. Facility health workers provide all available types of FP methods in Malawi.

Evidence about the quality and extent of rollout of the FP aspects of these programs in community and facility settings is sparse, which has led to calls for evaluation.

In response, the Malawi National Evaluation Platform (NEP) developed a multi-faceted set of analyses and data collection activities across the impact chain to evaluate FP programs in Malawi, with a focus on youth. NEP is a country-led and country-owned approach to evaluating the effectiveness of health policies and programs in four countries: Mali, Malawi, Mozambique and Tanzania [10]. The project also aimed to build public-sector institutional capacity in evaluation and analytical methods. In Malawi, it is led by the National Statistical Office (NSO), guided and supported by a high level advisory committee chaired by the Ministry of Health (MoH), and supported by a technical task team (TTT) that includes NSO, MoH and other relevant technical staff members as well as evaluation and capacity building experts at the Johns Hopkins Bloomberg School of Public Health (JHSPH) [10,11].

This paper describes the implementation strength assessment (ISA), which was carried out by NEP and analyzed by the TTT. The ISA was conducted to measure how well YFHS and FP programs are being implemented by identifying strengths and weaknesses that MoH can address to improve family program delivery. Implementation strength is quantity of a program delivered to a population [12,13], as distinct from the amount of a program received (coverage) and restricted to the structural quality aspects of a Donobedian framework [14]. The domains of ISA include: 1) Accessibility of services 2) Training of health workers 3) Supervision 4) Demand generation and 5) Availability of equipment and supplies [12].

The aim of this paper is to describe the strength of youth friendly health services, and all-age FP programs in Malawi in order to inform program and policy improvements. This report is the product of the NEP Malawi TTT analysis.

## METHODS

### Evaluation design

The ISA was a cross-sectional survey of facilities and providers using mobile phone interviews for data collection. It was conducted from May-August 2017 in all of Malawi’s 28 districts (including Mzimba North and South as one district).

### Sampling

For this evaluation, we selected all Government Health centers and hospitals, facilities run by religious entities on behalf of the government (Christian Health Association of Malawi [CHAM]), and NGO-run static clinics (funded by Banja La Mtsogolo, Population Services International [PSI], and Family Planning Association of Malawi) across all districts in Malawi. For each facility catchment area, we interviewed the facility In-Charge nurses (IC), all of the other facility-based health providers, all of the CBDA and a sample of 85% of the HSA.

### Data collection

In May 2017 a validation study was conducted in 2 districts, for which study staff interviewed health care workers by phone, then in-person and documented any inconsistencies. Before the main evaluation using results from the validation study, minor adjustments were made to the instrument to improve internal validity of the questions and to remove those questions for which getting valid answers in the broader survey seemed unlikely. Then in July and August 2017 we recruited a larger team, trained/re-trained them, as applicable and conducted the survey by phone. The study teams for both phases of the study included staff from NSO, MoH (Reproductive Health Directorate), JHSPH and temporary experienced data collectors hired just for this purpose.

**Figure 1:**
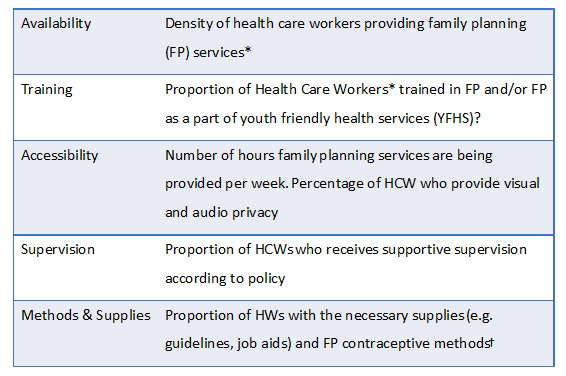
Domains of the implementation strength assessment (ISA). *HCW – Health Care workers include: HFW – health facility workers, HSA – Health Surveillance Assistants, CBDA – community-based distribution assistants. †Applicable to each type of HCW.

Topics from all domains of implementation strength (**Figure 1**) were included in the survey. It was written in English and translated into Chichewa and Chitumbuka before pre-testing. Data were collected on a tablet using Open Data Kit (ODK) software [15].

We first interviewed IC, obtaining contact information for FP providers in their facilities and the catchment areas. Then we collected information about FP services at that facility according to the ISA domains (**Figure 1**). Finally, we interviewed facility and community-based health care providers to ask more question on the same topics. Surveys took approximately 30 minutes.

### Data management and analysis

Responses were entered directly into tablet computers using electronic forms developed on ODK and were uploaded to the secure server when teams had internet access. The data were monitored for quality and completeness throughout the study and identified errors were corrected in real time.

We used 2008 Population and Housing Census projection [16] to obtain number of women aged 15-49 in each district to use in our calculation of provider density.

As part of the NEP’s capacity building activities, a data analysis workshop was held in Zomba in December 2017. It was attended and facilitated by the NEP TTT, which includes the MoH, NSO and some partners (eg, Save the Children) and was expanded to include additional FP stakeholders, including PSI and CHAM. Working in small teams, this expanded TTT group developed research questions and analyzed the data. The data were analyzed and results were displayed using Stata [17] and StatsReport (statsreport.io [18]), a web-based analysis and display interface. Graphs were created in excel using the frequencies and proportions generated by StatsReport. Maps were generated using R [19], StatsReport and ArcView ([20]).

### Ethics

The study was approved by JHSPH Institutional Review Board and the Malawi National Health Science Research Committee.

## RESULTS

### Background characteristics

**Figure 2:**
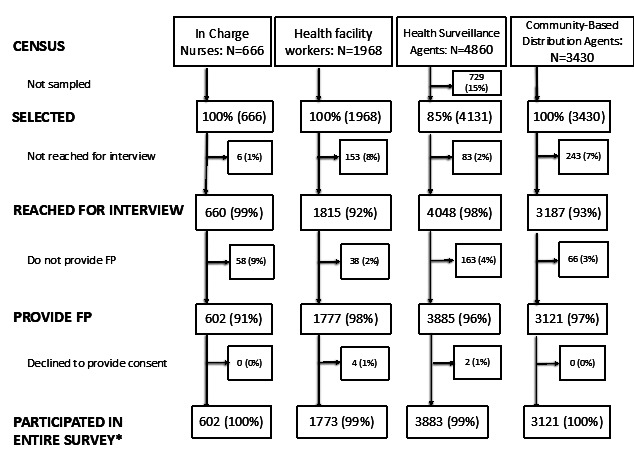
Health facility and community health worker population, selection, reached for interview, participated in entire survey. *Those who responded “no” to providing FP were not asked any additional family planning (FP) questions.

We interviewed health facility IC at 660 of 666 health facilities in Malawi (**Figure 2**). Of the facilities participating, 58 did not provide FP services. Most (95%; 55) of these facilities were CHAM facilities, and non-provision of FP was in accordance with their policies. However, 93% (51) of CHAM facility ICs who informed data collectors that they do not provide FP, also indicated that HSAs and CBDAs in their catchment areas do provide FP. We reached 1662 of 1815 (92%) HFWs, 4048 of 4131 (98%) HSAs selected, and 3187 of 3430 (93%) CBDAs for interview. Less than 10% of each HW cadre stated that they did not provide FP and less than one percent of those reached declined to participate.

The median age for health workers interviewed was in the mid 30s (range = 32-38 years) across health worker types (**Table 1**). More than two thirds of all types of workers reported being non-Catholic Christian. HSAs were more likely to be male (67%) while health facility workers (HFWs) were more likely to be female (68%) and CBDAs were more evenly split between the genders (48% male). HFWs and HSAs had higher levels of education than CBDAs. Compared to CBDAs (47%), a much higher proportion of HFWs (78%) and HSAs (95%) reported having been working in their catchment areas since or before January 2016.

**Table 1:** Background characteristics of health workers reached for interview, by cadre*

Number (%)	HFW 1815 (100%)	HSA 4048 (100%)	CBDA 3187 (100%)
Age (median, years)	35	32	38
Male gender	580 (32.7)	2594 (66.7)	1484 (47.8)
Religion:			
-Catholic	354 (22.0)	902 (25.6)	680 (23.9)
-Non-Catholic Christian	1227 (76.2)	2451 (69.9)	1880 (66.6)
-Muslim	29 (1.8)	160 (4.5)	270 (9.5)
Marital status (%):			
-Unmarried/Not in union	450 (27.2)	489 (7.9)	407 (13.7)
-Married/In union	1202 (72.8)	3472 (92.1)	2574 (86.3)
Education level (%):			
-Secondary school or less	119 (16.6)	1701 (44.3)	2485 (82.1)
-College certificate or more	597 (73.4)	2142 (65.7)	541 (17.9)
Worked in area since at least Jan 2016	110 (77.5)	485 (94.7)	55 (47.4)

HFW – health facility worker, HSA – health surveillance assistant, CBDA – community-based distribution agent

*Included all health workers reached for interview including those HWs that do not provide FP.

### Training

**Figure 3:**
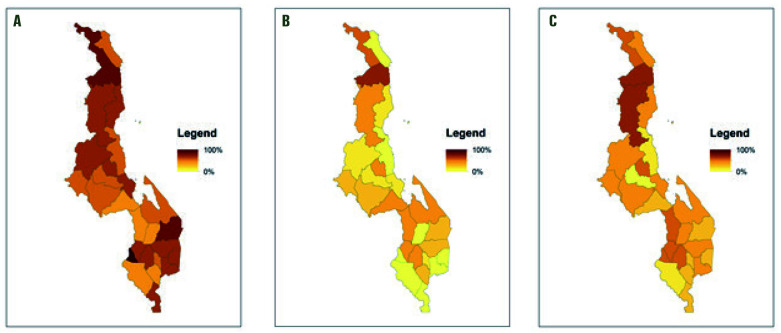
Availability of selected commodities on the day of interview by cadre by district. **Panel A.** Percentage of Health facility workers who had stock of male condoms, oral contraceptive pills, injectables & implants. **Panel B.** Percentage of Health Surveillance Assistants who had stock of male condoms, oral contraceptive pills and injectables. **Panel C.** Percentage of community based distribution agents who had stock of male condoms and oral contraceptive pills.

Just under half of all types of health care workers reported being trained in all methods they are authorized to provide (**Table 2**). This is similar to the proportion of HFW (43%) and CBDA (51%) reported having ever been trained in youth friendly health services (YHFS), but higher than the 26% of HSA who reported being trained in it. **Figure 3** shows these data by district.

**Table 2:** Implementation strength by domain and health care worker type

N (%)	Facility Data reported by In-Charge Nurse 660 (100%)	Health Worker Type		
**HFW 1815 (100%)**	**HAS 4048 (100%)**	**CBDA 3187 (100%)**
**Training of Health care Workers**				
Trained in all methods* in prior 2 years		786 (43.3)	1751 (43.3)	1483 (46.5)
Ever trained in YFHS		787 (43.4)	1065 (26.3)	1631 (51.2)
**Supervision:**				
Has supervision checklist that includes Youth FP	310 (47.1)			
Supervised for FP in prior 3 months†	503 (76.3)	1007 (55.5)	1997 (49.3)	1758 (55.2)
Last supervision covered youth FP topics		753 (41.5)	1576 (38.9)	1903 (59.7)
**Contraceptive methods and supplies:**				
Provides all FP methods*	525 (79.5)	1336 (73.6)	1978 (48.9)	2761 (86.6)
All FP methods* available on day of interview	407 (61.7)	1041 (57.4)	1160 (28.7)	1453 (45.6)
Has FP guidelines and job aids	518 (78.6)	1468 (80.9)	3219 (79.5)	2481 (77.8)
Has youth FP guidelines	380 (57.6)	989 (54.5)	1974 (48.8)	2014 (63.2)
Provides FP methods branded with social marketing	290 (44.1)	872 (48.0)	1648 (40.7)	1340 (42.0)
**Demand generation activities:**				
Conducted youth event in prior 3 months	272 (41.2)	689 (38.0)	2125 (52.5)	2110 (66.2)
Conducted SRH talks in prior 3 months			2121 (52.4)	2645 (83.0)
Conducted youth spaces in prior 3 months	273 (41.4)	855 (47.1)	1935 (47.8)	2105 (66.0)
Conducted community meetings in prior 3 months	281 (42.6)	610 (33.6)	2924 (72.2)	2617 (82.1)
Facility has peer educators for FP	257 (38.9)			
**Accessibility:**				
Has special days for youth FP	203 (30.8)		980 (25.1)	1858 (59.5)
Conducted mobile outreach in prior 6 months	398 (60.3)		2330 (59.8)	1164 (47.8)
Ensures privacy during FP consultations‡	532 (80.6)	1262 (69.5)	2358 (58.3)	2031 (63.7)
Provides FP the minimum hours per week§	325 (49.2)		2501 (61.8)	1344 (42.2)

HFW – health facility worker, CBDA – community-based distribution agent, FP – family planning, SRH – sexual and reproductive health, YFHS – youth-friendly health services, OCP – oral contraceptive pill

*Appropriate to HW type. HFW: counseling, condoms, OCP, injectables, implants; HSA: counseling, condoms, OCP, injectables; CBDA: counseling, condoms, OCP.

†For facilities this is by someone external to the facility.

‡For facilities, must have a private room.

§For facilities: ≥24 h/week of access. For CBDA/HSA: ≥12 h/week of access.

### Supervision

About half of all respondents reported having received supervision for FP in the prior 3 months and three quarters (76%) of facility staff reported receiving FP supervision from someone external to the facility (eg, district authority) in the 3 months preceding the survey. But less than half (47%) of facility staff reported having had a supervision visit that focused on youth FP over this time period (**Table 2**).

### Family planning methods & supplies

**Figure 4:**
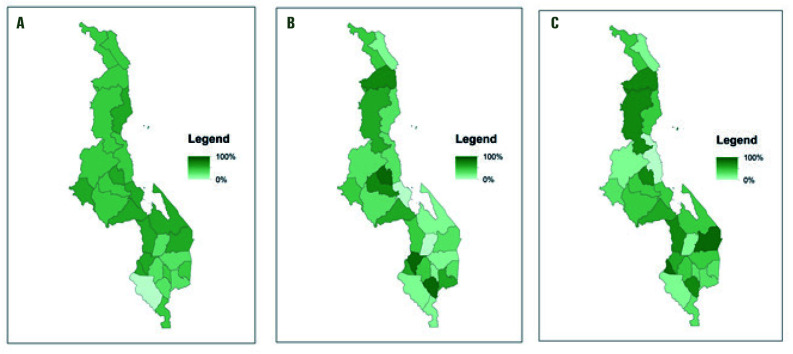
Proportion of health workers trained in family planning (FP) in the prior 2 years, by cadre and district. **Panel A.** Health facility workers. **Panel B.** Health surveillance assistants. **Panel C.** Community based distribution agents.

A large proportion (80%) of health facility IC reported that their health facilities provide all FP methods they are authorized to provide. While 74% of HFWs reported providing all the methods they are authorized to provide, only 62% had these methods available on the day of interview. While 49% of HSAs reported providing all methods they were trained to provide (condoms, OCPs, injectables), only 29% had these methods available on the day of interview. While 87% of CBDAs reported providing their authorized methods (condoms and OCPs), less than half (45%) had these methods available on the day of interview. **Figure 4** shows stockouts per district.

While nearly 80% of respondents of all worker types reporting having both FP guidelines and job aids, a much lower proportion reported having guidelines specific to youth FP (**Table 2**).

### Demand generation

Respondents were asked if they had organised or assisted with demand generation activities for FP such as youth events, door-door health talks, community meetings on youth accessing HIV ad FP counselling and social marketing past in the prior 3 months (**Table 2**). About three quarters of HSA and CBDAs said they had. HSAs said they had participated in community meetings to promote youth to get HIV testing and FP counselling. More than half of CBDAs (66%) and HSAs (53%) said they had organised or assisted with youth events in the prior 3 months. About the same proportions reported having participated in youth-oriented spaces such as youth clubs or youth centres. Most CBDAs (82%) said they had gone door-to-door in your community to deliver health talks on sexual and reproductive health, HIV prevention, and FP to youth and slightly over half of HSAs (52%) reported these activities.

### Accessibility

**Figure 5:**
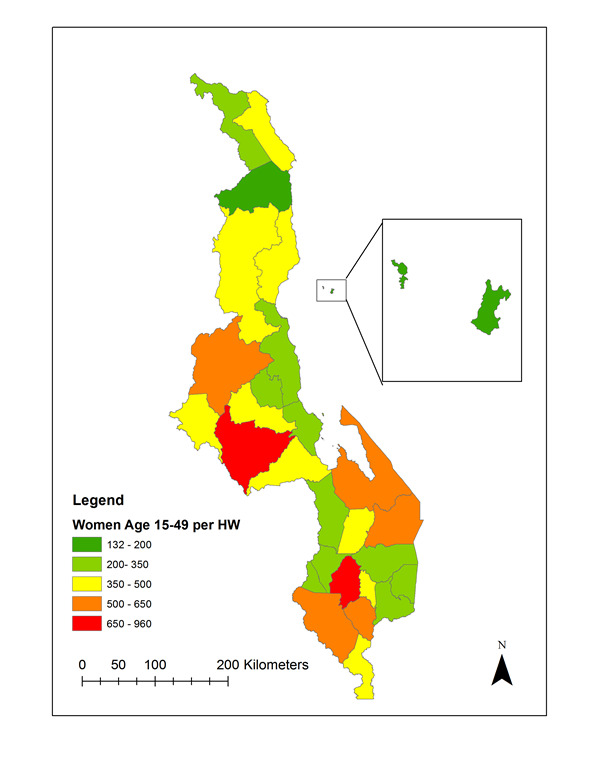
Health worker density: number of women aged 15-49 per family planning provider interviewed, by district.

We compared the number women 15-49 years of age to the number of FP providers by cadre across all districts. Lilongwe and Blantyre have the lowest client-provider ratio while Likoma and Rumphi have the highest provider-client ratios, respectively (Figure 5).

Most health facility staff (81%) reported ensuring privacy during FP consultations, although HSAs (58%) or CBDAs (64%) were less likely to report ensuring privacy. While nearly three quarters of CBDAs stated they have special days for FP, fewer ICs and HSAs reported having them. More HSAs (62%) reported provide FP the minimum hours per week (at least 12 hours a week) than CBDAs (42%). Less than half of all facilities (49%) reported providing FP the minimum 24 hours per week. Slightly under half (48%) of CBDAs and 60% of both HSAs and facilities (60%) reported participating in outreach clinics that provide family services to hard to reach areas since 2016.

## CONCLUSIONS

Despite having been on the forefront of YFHS strategy development, health workers in Malawi face barriers to providing consistent and comprehensive FP services, especially those targeted to youth.

This evaluation was conducted as part of the NEP in Malawi, from design through analysis and reporting. Over the 4 years of the project, on subjects as varied as modeling childhood illness and nutrition, data quality assessment, qualitative analysis and using routine data to estimate pneumonia incidence, the platform has enabled the NSO, MoH and other key stakeholders to work as teams to analyze the data and prepare and present results to policy makers. This manuscript reflects the results of analysis by the TTT in December 2017.

Our study sample included all health care providers of FP in Malawi, one of the largest surveys of its kind. Results include a comprehensive assessment of the implementation strength of the FP services, in general, and targeting of youth, in particular. Assessing the quality of services provided to youth has been a challenge but its importance has been more widely recognized in recent years [21]. Many different components of the health system need to work in synchrony for a successful scale up of youth friendly services [22-24].

About 1 in 4 facility workers and half of the HSAs do not provide all methods they are expected to provide and even fewer had all the methods available on the day of the interview with considerable variation by district. Based on these results, it appears to be difficult for health workers and facilities to stock all required commodities. This was true particularly of the HSA, but also of the other cadres. This system weakness may limit the methods women and young girls can choose and result in more frequent switching of methods [25,26]. Women and young girls may experience frustration when they try to access reproductive health services because they are available less often than the minimum hours/week, in particular among CBDAs and facilities. Nearly 2/3rds of HSAs provide services the minimum hours a week, which may be filling gaps, but is still far from full access.

Less than half of all provider cadres reported received training in FP in the prior two years, and similar proportions of facility workers and CBDA reported ever receiving training on YFHS. Only a quarter of HSA reported having received training on YHFS, suggesting an additional weakness in the youth focus. Given innovations in methods (implants), misconceptions about side effects [27], and the need to address concerns about confidentiality, stigma and societal pressures around contraception [27], plans and support for training of health workers on FP and YFHS should be re-evaluated. As a caveat, it is possible that incentives for attending training coupled with true interest in learning may have biased responses. We tried to validate the training records, but were unable to identify any source of data to corroborate health worker responses. To address the lack of an available data source, we recommend that standard training records be kept and documented in a database that can be made available to both government and partner organizations.

Approximately half of all cadres were supervised for FP every 3 months, which is the timeline required for supervision per MoH policy. Also, although the majority of CBDA received supervision on youth topics, it was less common for HSA and HFW to receive that support. Improving coverage and quality of supervision and training can improve the capacity of providers and increase effectiveness [28].

Other research has suggested that to reach youth, special days, times and approaches are necessary [29,30]. In this study despite most facilities reporting that they provide youth friendly health services, only half report providing special days for youth. Youth-specific FP guidelines or protocols were available in very few facilities that provide these services, and about only a third of respondents reported having special rooms for youth-focused services. Without appropriate supervision, guidance, specific mentorship focusing on serving youth, and special services for them, it will be hard for Malawi to address their special needs.

CBDA handle most of the youth and sexual and reproductive health events and talks but are the least educated and most connected with community, so youth may feel the risk of disclosure to be higher. CBDAs report holding special days for youth services but facilities and HFW seem to rarely offer these services. If youth favor CBDAs because of their ability to offer special services to them, their choices are limited to condoms and pills, missing out on the option to use injectables, implants or LACs.

While these results reflect a snapshot captured in mid-2017 and does not compare results with implementation strength before YHFS was implemented, it includes all districts and nearly all facilities in the country. The sample size was calculated to be representative at district level for HSAs and it was a census of Health facility workers and CBDAs. While it was not able to evaluate every aspect of the program, and did not address quality of care, topics of the evaluation covered a wide range of supply-side indicators that estimate the readiness of the health systems to provide FP, especially to the youth. Calling health workers by mobile phone to ask them questions was innovative and reduced the burden and cost of data collection, especially given the fact that we surveyed the entire country. However, we were unable to validate all indicators during the field-based pilot, and because most indicators were self-reported for some responses there is still some uncertainty about bias and validity.

### Policy implications

We hope the Reproductive Health Directorate will use the findings to make recommendations. Here are some that the authors suggest be considered:

1. Reinforce the commodity supply system so that all facilities and workers have a consistent supply of all contraceptive methods.
2. Increase, if possible, the number of hours that FP is actually being offered in facilities and by community health workers – at least to the minimum required number of hours
3. Restock FP guidelines and IEC materials at all service delivery points
4. Review training records for all healthcare workers providing FP, especially HFWs and HSAs. Consider retraining on injectables, implants, and YFHS.
5. Redouble efforts to ensure frequent supportive supervision involving mentoring, coaching and reporting (on service delivery and stocks levels) to improve quality service delivery
6. Establish special rooms and days for youth activities to provide better FP service access to youth.

These recommendations are meant for the entire country, although as we have shown, these data can be used to evaluate the program at a more granular level (eg, district). The data should be analyzed and mapped, to facilitate use by district program officers to show areas of weakest (and strongest) implementation by indicator and domain. National and subnational program managers can use StatsReport to produce these maps. They can be used to target resources and focus to areas with weaknesses in specific strength domains.

The good news is that the barriers identified are surmountable. In light of great success in raising mCPR from in the context of similar if not the same barriers, with recommendations herein, we have a great opportunity to accelerate improvements in FP outcomes and impact.
